# Congenital Aniridia: Clinic, Genetics, Therapeutics, and Prognosis

**DOI:** 10.1155/2014/305350

**Published:** 2014-10-29

**Authors:** Pedro Calvão-Pires, R. Santos-Silva, F. Falcão-Reis, A. Rocha-Sousa

**Affiliations:** ^1^Faculty of Medicine, University of Porto, Hospital de São João, Alameda Professor Hernâni Monteiro, 4200-319 Porto, Portugal; ^2^Department of Sense Organs, Faculty of Medicine, University of Porto, Hospital de São João, Alameda Professor Hernâni Monteiro, 4200-319 Porto, Portugal

## Abstract

Congenital aniridia is a rare condition related to a deficiency in the PAX6 gene expression, which may occur as a result of a family inheritance or a sporadic occurrence. Additionally, this condition may occur as an isolated ocular phenotype or in association with a systemic syndrome. The most common abnormality is iris hypoplasia; however, a panocular disease which also affects the cornea, anterior chamber of the eye, lens, and the posterior segment with presence of optic nerve and foveal hypoplasia is also evident. The development of keratopathy, glaucoma, and cataract is frequent and its presence has implications in the patient's visual acuity. Managing aniridia is challenging since the focus is on treating the previously mentioned disorders, and the outcomes are often disappointing. In this paper, we shall review the epidemiology, pathophysiology, and clinical characteristics of patients with aniridia. We shall also make a review of the therapeutic options for the several conditions affecting this syndrome and consider the genetics and prognostic factors.

## 1. Introduction

Congenital aniridia is a rare condition (incidence between 1 : 64000 and 1 : 100000) that typically affects both eyes and is associated with PAX6 gene mutations. PAX6 gene is located in the short arm of chromosome 11 (11p13). This gene plays a role in the development of eye, mainly in the development of the cornea, iris, lens, drainage angle of the eye, and ciliary body [1–4].

Two-thirds of aniridia cases are inherited in an autosomal dominant pattern and 1/3 are sporadic cases. Moreover, sporadic cases may affect only the eye or be associated with various systemic disorders, as is the case of the association with Wilms tumour, resulting in the WAGR syndrome (Wilms tumour-Aniridia-Genital anomalies-Retardation), which is the most common and the most important aniridia associated syndrome [1–4].

The name “aniridia” derives mainly from the iris hypoplasia, the most evident manifestation, although it ranges from almost total to only a mild phenotype. However, there are other ocular manifestations in the different eye segments that are associated with aniridia, namely, from anterior to posterior, the cornea with dry eye and keratopathy, glaucoma and anomalies of the drainage angle of the eye, lens opacities and lens subluxation and retinal, and macular and optic nerve disorders associated with nystagmus. Patients also show strabismus and visual acuity (VA) reduction, with bad visual prognosis since early age [1, 2, 4, 5].

## 2. Materials and Methods

A review of the literature found on* Pubmed* database, between 2004 and 2014, using “aniridia,” “keratopathy,” “Boston keratoprosthesis,” “glaucoma,” “foveal hypoplasia,” and “WAGR syndrome” as keywords, as well as suggested and cited articles that proved relevant, was carried out.

Papers written in English, French, and Portuguese were selected, based on the title and abstract. Papers on any other language or related only to acquired aniridia were excluded.

## 3. Results and Discussion

### 3.1. Epidemiology

The incidence of congenital aniridia is between 1 : 64000 and 1 : 100000 and there is no clear association with gender or ethnicity. Two-thirds of aniridia patients have an affected parent. The majority of hereditary cases have an autosomal dominant inheritance, with complete penetrance. However, there are differences in the phenotype expression.

Sporadic aniridia cases account for 1/3 of all cases, 2/3 of which present a new mutation that will be inherited by future offspring in an autosomal dominant way [1–3, 6].

### 3.2. Genetics

Classic aniridia presents a mendelian inheritance pattern and is an autosomal dominant disease, which happens due to a loss of function of one of the copies of PAX6 gene. It is detectable in 90% of the cases, resulting from genic mutations in 2/3 and from chromosomal rearrangements in 1/3 [3, 6].

PAX6 gene has 14 exons and is located in the short arm of chromosome 11 (11p13), codifying a transcriptional regulator with two DNA binding places (paired domain and homeodomain) and a transcriptional transactivating domain [3, 6].

The gene is expressed in the developing eye during the foetal age and there is need for both copies of PAX6 gene to be present for the normal eye to develop. It codifies a regulator protein essential for the processes involved in the genesis of the cornea, iris, lens, drainage angle of the eye, ciliary bodies, and all the retinal layers. It is also important for the development of some cerebral regions, spinal cord, olfactory tract, cerebellum, and alfa-pancreatic islets differentiation. After birth and during the life time, it is also expressed in the eye (playing an important role in the control of corneal epithelium proliferation), cerebellum, and pancreas (regulates alfa-cells activity) [3, 6].

As of 17 March 2014, the total number of unique DNA variants reported in the* Leiden Open Variation Database *for the PAX6 gene (http://lsdb.hgu.mrc.ac.uk/home.php?select_db=PAX6) is 357. The majority of the mutations are associated with a loss of function of PAX6, generally microdeletions or deletions that produce a stop codon (nonsense), splice mutations, or mutations which change the reading pattern of the gene (frameshift) and generate nonfunctioning or truncate proteins [6].

Approximately, 10% of PAX6 mutations are the missense type, resulting from the alteration of one amino acid into another. These mutations normally present a milder aniridic phenotype [6].

It is worth mentioning that there are reports of 3 children with two mutated copies of PAX6, one inherited from each parent. Two of these died early after birth presenting anophthalmia and brain anomalies and the other survived but presented microphthalmia and microcephaly [3].

Aniridia can also develop due to chromosomal disturbances that affect totally or partially the PAX6 or the contiguous ELT4 gene. ELT4 is a gene with transcriptional regulators of PAX6. If the regulators are deleted or separated from the PAX6's transcriptional unit by deletion, inversion, or translocation, the outcome is also aniridia [3]. It is important to bear in mind that there is this regulator unit 200 kb after the PAX6 gene codification site in cases such as apparent mutation-free aniridia with familiar inheritance since there can be chromosomal rearrangements and mutations that affect this control unit without affection of PAX6 itself [6].

Almost 1/3 of sporadic aniridia cases have PAX6 deletion and deletion of another contiguous gene, the WT1 gene (these two are separated from each other by 700 kb in 11p13). With deletion of both genes, the outcome is WAGR syndrome, so called because it occurs with Wilms tumour, aniridia, genitourinary tract defects, and mental retardation. Should the other WT1 gene of patients with this syndrome lose its function due to somatic loss, the development of nephroblastoma/Wilms tumour (most frequently) or gonadal tumours (rarely) can occur. Thirty-six percent of bilateral Wilms tumours appear in association with aniridia. Yet, since the cells susceptible to malignant processes are only present until childhood, it is uncommon for Wilms tumour to develop after the age of 8 [1, 3, 6]. There is also an association of WAGR syndrome with obesity, a situation called WAGRO syndrome (“O” for obesity). This syndrome is characterized by the onset of obesity at early ages, polyphagia/hyperphagia, and high cholesterol levels. The deletion associated with this syndrome is a larger 11p deletion with affection of BDNF gene [1, 7].

There are other syndromes associated with aniridia [6]:

- Gillespie syndrome, with autosomal dominant inheritance, also caused by PAX6 gene mutations and which comprises ataxia and mental retardation;
- Peters syndrome caused by mutations involving PAX6, PITX2, CYP1B1, or FOXC1 genes. It generally has autosomal recessive inheritance and is associated with anterior chamber malformations such as corneal opacities, posterior stroma, and Descemet's membrane absence and adhesions between posterior cornea and iris/lens;
- Rieger type I syndrome caused by mutations involving PITX2 or FOXC1 genes. It has an autosomal dominant inheritance and presents malformation of the anterior segment of the eye; 50% of cases present glaucoma with vision loss and systemic alterations.

In aniridia cases, a direct correlation between genotype and phenotype has not been established yet. There are many clinical variations and patients with the same mutation can present different phenotypes. Moreover, there is also phenotypic variability between affected members of the same family. However, there is an apparent relation between the type of alterations in the protein and the ocular presentations, as patients with confirmed mutations that introduced premature stop codons seem to present phenotypes typical of aniridia, whereas patients with missense mutations have more nonaniridia disorders [6].

### 3.3. Clinical Presentation

#### 3.3.1. Cornea

Aniridia associated keratopathy (AAK) occurs in approximately 20% of aniridia patients. However, changes in the ocular surface can be found in up to 90%, being one of the manifestations associated with aniridia that worsens vision even further [1, 4].

Keratopathy occurs mainly due to a deficiency in corneal limbal stem cells. There are other factors contributing to the keratopathy such as a poorly differentiated corneal epithelium, abnormal cellular adhesions, impaired response to lesions, and corneal conjunctival infiltration, all contributing to an impaired environmental surrounding of the corneal limbus [1].

The first signs of AAK start developing in the first decade of life with the appearance of irregular thickening and neovascularization of the peripheral cornea, which with time progresses to invasion of the centre and finally to the involvement of the whole cornea. As a result, recurrent erosions, ulceration of the cornea, and subepithelial fibrosis become evident. This leads to corneal instability and opacification, stromal scars, and increasing central corneal thickness [1, 4]. Brandt et al. observed an increase of central corneal thickness of at least 100 *μ*m, which is superior to what is considered normal [8]. Corneal opacification can occasionally be the first presenting sign of aniridia [2].

Surgeries that involve manipulation of the limbus and application of topical antimetabolites, used during glaucoma surgeries, are identified as keratopathy deteriorating factors [2]. There can also be asymmetrical corneal lesions in patients with aniridia, possibly owing to traumatic or surgical events [4].

Genetically, PAX6 seems to play an important role in corneal development influencing the proliferation, differentiation, and cellular adhesion. In mice heterozygous for PAX6 gene, the corneal epithelium is thinner and weaker. It is also infiltrated by goblet cells which correspond to corneal infiltration by conjunctiva cells (conjunctivalization of the cornea) and reflect limbal stem cell deficiency. PAX6 also regulates expression of two cytokeratins (3 and 12) as well as the expression of some adhesion molecules like desmoglein, *β*-catenin and *γ*-catenin. In aniridia, all these molecules have a reduced expression and there is fragile corneal epithelium with cell vacuolization and abnormal spacing [1, 4]. On the other hand, there is also a defect of the glycoconjugate from the corneal cells' surface responsible for restricting cell's migration capacity. It is the accumulation of all these factors that originates recurrent erosions and ulcers [1].

PAX6 also regulates the expression of matrix metalloproteinase 9 (MMP-9) or gelatinase-B in the cornea. Matrix metalloproteinases are responsible for degrading collagen in cell remodelling and healing processes in the cornea. So, when erosions and ulcers occur, these processes are impaired and there is an increased cell apoptosis and accumulation of fibrin. Loss of corneal transparency with corneal opacification and infiltration by inflammatory cells in response to IL-1 are then a consequence of the above-mentioned factors with the occurrence of simultaneous stimulation of corneal neovascularization [1, 4].

It is important to characterize the observed pathological corneal changes for each patient as treatment varies accordingly. Central corneal thickness, superficial neovascularization, subepithelial fibrosis, opacities, and corneal irregularities can be found. Clinically, these are expressed as persistent and recurrent epithelial defects, conjunctival infiltration, limbus hyperplasia, chronic inflammation, conjunctival hyperemia, and keratinization. The typical signs of the keratopathy are dry eye, red eye, photophobia, epiphora, VA reduction, and blepharospasm, with increased risk of bacterial infections and eye perforation [1, 4]. Abnormal tear film with a reduced tear breakup time and reduced tear meniscus can be present too [1].

Ocular surface instability associated with inefficient corneal epithelium regeneration can explain why dry eye and loss of corneal protection barrier are encountered. Furthermore, recurring lesions of the ocular surface initiate a process of squamous metaplasia. This process, which is reversible, happens before the pathologic keratinized epithelium develops. This transformation is easily observed microscopically and it is possible to identify this squamous metaplasia using impression cytology, which can provide a quicker diagnosis and a better treatment [1, 4].

**Figure 1:**
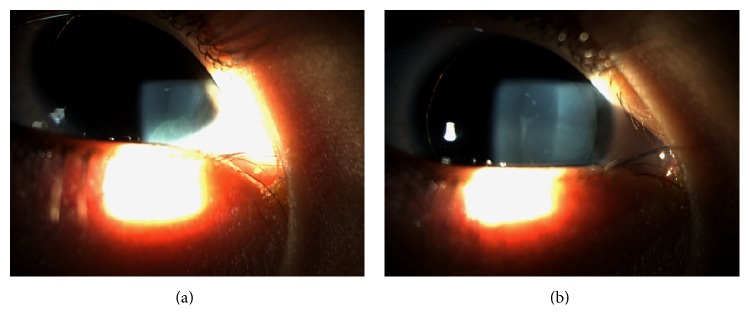
Mild keratopathy in patient with limbal insufficiency.

**Figure 2:**
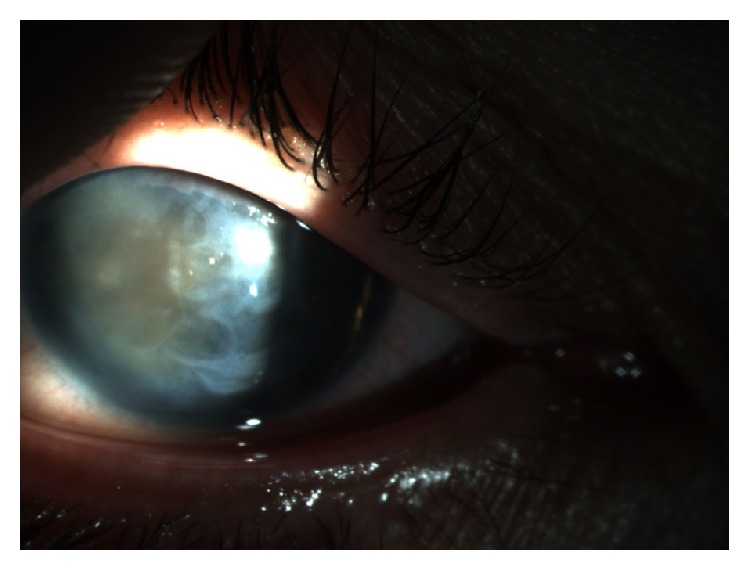
Anterior segment of patient with aniridia at retroillumination.

In order to define a specific treatment for each patient, López-García et al. [4] proposed a classification for the keratopathy associated with aniridia in 4 phases:

 phase 0: patient with subclinical limbal insufficiency, already with corneal degenerative processes but without expressing clinical signs;
 phase 1: patient with slight limbal insufficiency, that refers a maximal of 2 corneal ulcers or erosions in the past 6 months, slight photophobia, and epiphora and also slight vascular pannus not exceeding 1 mm from the limbic arch and small disorders in the absorption of fluorescein (Figures 1(a) and 1(b));
 phase 2: patient with moderate limbal insufficiency that refers to 3 or more corneal ulcers or erosions in the past 6 months and exhibits permanent instability of the lacrimal film and vascular pannus (with or without subepithelial fibrosis), which involves at least the peripheral half of the cornea. Photophobia, epiphora, and red eye are a constant in these patients;
 phase 3: patient with severe limbal insufficiency that exhibits central vascularization of the cornea with permanent clinical signs such as photophobia, epiphora, and red eye and loss of vision because of central corneal involvement (Figure 2).

*Treatment*. The therapeutic approach to the patients will depend on the degree of lesion shown by the ocular surface. For patients in phase 0 or 1, the keratopathy can be managed simply with artificial tears without preservatives, such as sodium hyaluronate preparations. On the other hand, most of the preparations used for the treatment of glaucoma have preservatives and can contribute to further corneal dysfunction. It is also important that these patients use dark glasses for relief of photophobia symptoms and that they use ocular lubricants and topical antibiotics for the treatment of corneal erosions (as occurs in a patient without keratopathy) [4].

For patients with keratopathy on phase 2, the treatment requires autologous serum or amniotic membrane transplant, which will reinforce the limbo-corneal surface. Autologous serum has many factors that promote proliferation, migration, and corneal epithelium differentiation. Amniotic membrane transplant has various mechanical and biological properties that make it a good therapeutic choice for the treatment of partial lumbar insufficiency. It improves the environment of the extracellular matrix of lumbar epithelial cells and enhances the expansion and survival time of the cells. However, this treatment provides only temporary results with the recurrence of symptoms happening in time [4].

For patients with keratopathy on phase 3 who exhibit severe limbal insufficiency, the treatment involves supplying lumbar cells by means of limbus transplant. As aniridia most commonly affects both eyes, an autologous transplant is not viable. The two alternatives in these cases are allografts with tissue from healthy relatives having high HLA compatibility or from cadavers. Penetrating keratoplasty alone has a poor prognosis due to the frequent recurrence of lesions similar to the pretransplant state, followed by rejection of the allograft. This frequently takes place in the first year after surgery. On the other hand, homologous lamellar limbo-keratoplasty, a procedure that combines limbus transplant with amniotic membrane transplant, shows beneficial outcomes for the treatment of these patients. The association of this procedure with systemic immunosuppression achieves even higher success rates [1, 4, 9, 10].

**Figure 3:**
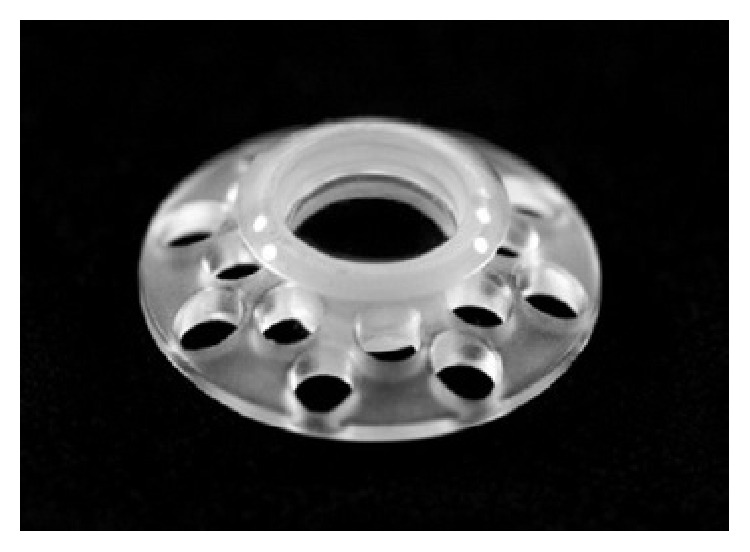
Boston type I keratoprosthesis (image rights to Claes H. Dohlman M.D., Ph.D.).

So as to circumvent the problems related to the previous treatments and their failure, implantation of artificial cornea prosthesis is being implemented more frequently. This procedure substitutes the central cornea by a Boston type I keratoprosthesis, a cylinder made up of nonimmunological material (Figure 3). This device overcomes the allograft rejection problem (and also the need for systemic immunosuppression), the neovascularization, and the conjunctival infiltration. Other advantages of this prosthesis are its anterior spherical curvature, which helps to maintain a good curvature of the eye allowing an immediate improvement after the implantation as well as the fact that it is built to match the eye's axial length. The disadvantages of the keratoprosthesis are severe, including bacterial endophthalmitis, corneal tissue melting, and prosthesis extrusion. Nowadays, the endophthalmitis can be prevented and treated with the use of vancomycin and fluoroquinolones (and also third-generation cephalosporins, but they have a worse ocular absorption). Another frequent yet less severe complication is the formation of a retroprosthetic membrane. However, it has to be treated because of its contribution to VA deterioration. There are increasingly better success rates for this procedure and a decrease in reported complications, making this a more common approach for the treatment of keratopathy. Its classical indication is the treatment of the keratopathy when the risks are unacceptable for an allograft. Glaucoma is frequently reported to develop or worsen after implantation of the keratoprosthesis. The presence of glaucoma (before and/or after the surgery) is still a therapeutic challenge and it is also a limitation to the long-term visual outcome [10–12].

Some recent results reported with the keratoprosthesis are in Table 1.

#### 3.3.2. Glaucoma

Although glaucoma or ocular hypertension associated with aniridia has been reported in between 6% and 75% of patients, the risk of developing glaucoma is generally accepted to be around 50%. It usually develops during the first two decades of life. It is worth mentioning that congenital glaucoma in aniridia is rare [2, 13].

When associated with aniridia, the glaucoma's physiopathology is complex, owing to the abnormal development of the drainage angle of the eye which is obstructed and impairs the aqueous humor's flow through the Schlemm's canal. During the development stages of the eye and its anterior chamber, the iridocorneal angle does not have normal development. Moreover, there are also progressive angle changes during the first two decades of life. Since birth, the trabecular meshwork is open, without any structure blocking the normal drainage, yet with time there is development of adhesions between the remains of iris stroma and the angle wall (peripheral anterior synechiae) that might extend and obstruct the trabecular meshwork, thereby closing it. Iris rotation of the remainders can happen in association with all these changes [1, 13, 18]. Other mechanisms reported to contribute to the development of glaucoma are Schlemm's canal absence and angle closure preceded by miotic therapy [2].

The diagnosis of glaucoma and its classification is made via the observation of optic disk cupping or loss of retinal nerve fiber layer, the observation of the width of anterior chamber angle, the measurement of intraocular pressure (IOP) by means of ocular tonometry, and the examination of the visual fields. Although significant, the feasibility of all these exams is dependent on the cooperation of the patients [1]. Furthermore, it is necessary to bear in mind that possible error causes may be encountered. For instance, it was verified that patients with aniridia have an increased central corneal thickness, which is sometimes quite relevant. The importance of this increase is due to the error it introduces in tonometry, leading to a falsely increased IOP value. Patients that are diagnosed with ocular hypertension may simply have increased central corneal thickness when using only the tonometry results. It is essential to correct the IOP having in mind the central corneal thickness value (which is measured by pachymetry). The optic nerve and nerve fibre layer, as well as gonioscopy, are more accurate and important exams when diagnosing and assessing the progression of glaucoma [8, 19].

*Treatment*. The treatment of glaucoma is difficult and challenging and needs medical and surgical measures to achieve IOP control from the first manifestations and for the duration of the patient's life.

Medical treatment is based on the use of miotic eye drops with or without oral carbonic anhydrase inhibitors. As years pass it may become insufficient for some patients and a majority become refractory to its application [1, 13, 18]. However, it is the first choice treatment when glaucoma is diagnosed in older children and adolescents [19].

Surgical treatment includes procedures used for treating glaucoma (argon laser trabeculoplasty, goniotomy, trabeculotomy, trabeculectomy with or without associated topical antimetabolites, surgery with implantation of glaucoma drainage devices and cyclocryotherapy or cyclophotocoagulation) and a prophylactic procedure (prophylactic goniotomy) [1, 13].

The reported results of argon laser trabeculoplasty have not proved satisfactory [2, 13]. Therapeutic goniotomy, a procedure that dissects iris tissue adhesions that are connected to the trabecular meshwork, has shown poor success rates (between 0% and 20%) [13, 18, 20]. Trabeculectomy also shows unsatisfactory results with the need for re-intervention in many patients [2, 13, 20].

As for cyclodestructive procedures (cyclocryotherapy and cyclophotocoagulation), they are efficient in reducing the IOP but cannot be considered as first line treatments because they exhibit important complications like* phthisis bulbi* and retinal detachment (both appear in 50% of cases), progressive lens opacification and blindness [1, 13, 21, 22].

Trabeculotomy, which consists of removing the trabecular meshwork, has reported success rates ranging from 0% to 83%. The disadvantages of the procedure are the possibility of vitreous loss, scleral collapse, choroidal or retinal detachment and endophthalmitis [1, 13]. Adachi et al. [20] suggest that this should be the initial surgical procedure when treating a medically non-controlled glaucoma associated with aniridia. It appears to be more useful in younger patients because they have a higher prevalence of glaucoma with slight angle closure and with fewer adhesions between the iris and trabecular meshwork [13, 19].

Surgery for implantation of a glaucoma drainage device is very efficient in controlling IOP in glaucoma associated with aniridia with reported long-term success rates ranging between 66% and 100%. The implants used are the Ahmed valve or the Molteno and the Baerveldt tubes. One disadvantage of these implants is that they require intentional occlusion during a few weeks after surgery to avoid ocular hypotony. During that time, an intensive medical approach is needed to control the IOP [11, 13].

Some authors do not recommend the implantation of glaucoma drainage devices as an initial procedure because of its complications [1, 13]. Some reported complications include anterior chamber flattening, tube migration, and retinal detachment (associated with vitreoretinopathy and giant retinal tear) [23]. On the other hand, Arroyave et al. [23] had good results with them, thus suggesting that this procedure can be considered for the initial treatment of glaucoma.

Prophylactic goniotomy seems to be efficient for the prevention of glaucoma when early signs of angle changes are found and a success rate between 89% and 100% was recorded. This surgery tries to separate the pathological extensions of iris tissue from angle wall preventing its closure. New tissue adhesions do not occur later [1, 13]. Chen and Walton [13] reported very satisfactory results with the use of this technique. They performed prophylactic goniotomy in 55 eyes of 33 patients (one procedure in 19 eyes and two procedures in 36 eyes) with a mean of 200° extension for each eye. Forty-nine eyes had IOP control without medication and 6 had IOP control with association of a topical medication. They did not report failures in the mean follow-up time of 9,5 years.

Glaucoma control is specially challenging after a keratoprosthesis implantation. Firstly, it is difficult to evaluate the IOP and optic nerve lesion before the surgery due to corneal alterations in many patients. Secondly, the tonometry is of poor value in eyes with a keratoprosthesis, since the basic conditions for tonometry are not met. Digital palpation of the eye is the most rigorous method available. Thirdly, a lot of comorbidities are present in the eye with aniridia which limit the evaluation of the visual fields (even after surgery) [11].

Kamyar et al. [11] verified that the keratoprosthesis can in itself cause the development of glaucoma or worsen the preexisting glaucoma. They point out that the prosthesis can close the angle itself or help form peripheral synechiae that progressively close the angle. It also contributes to local inflammation. These authors also point out the difficulty in perceiving the eyes which are in danger of acutely developing elevated IOP and which will develop progressive late glaucoma. For prevention, they propose an aggressive treatment to control IOP including implantation of a Molteno or Baerveldt tube (before the keratoprosthesis or even during the same surgery) or cyclophotocoagulation.

#### 3.3.3. Iris and Lens

Approximately, 50% to 85% of patients with aniridia develop cataract, the most affected patients being adolescents and young adults. Cataract affecting children is rare, yet congenital lens opacities are common, resulting from foetal vascularization remains in the anterior lens capsule (tunica vasculosa lentis) or from the persistence of the pupillary membrane. These opacities may not contribute to VA worsening despite being present from an early age. Cataracts affecting adolescents are more frequently cortical, subcortical, or lamellar cataracts, whereas the congenital type is more frequently anterior or posterior polar cataracts [1–3, 24].

Frequently and due to its contribution to VA, cataract extraction is recommended with or without an intraocular lens implantation. However, it must be borne in mind that the surgery has complications, specially related to the lens capsule. When aniridia is present, the capsule is very fragile, as histological examination of the anterior capsule obtained from patients with aniridia and cataracts has already shown [1, 25].

Lens subluxation or positional changes may also occur but are less frequent [3].

**Figure 4:**
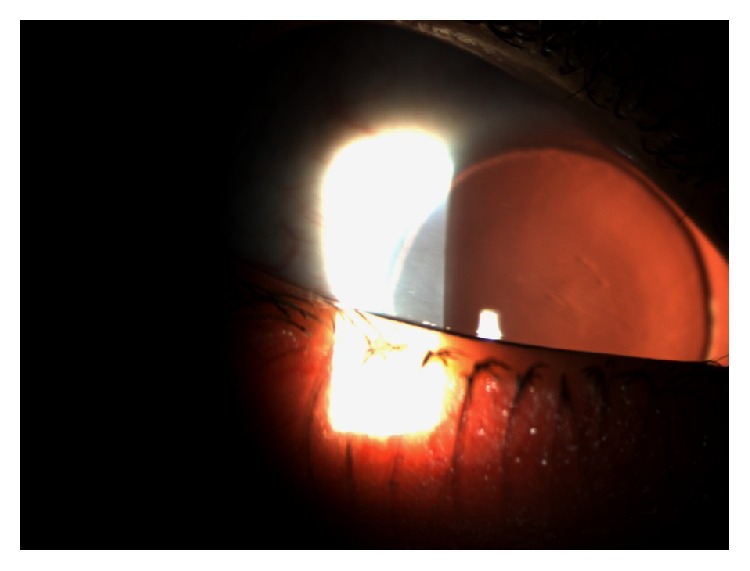
Anterior segment of patient with aniridia by retroillumination.

Iris hypoplasia, the most common finding in aniridia has different contributions to the disease's physiopathology like VA reduction, photophobia, and also aesthetical implications (Figure 4). At gonioscopy or histological examination, it is almost always possible to find iris tissue remains and it is considered that iris hypoplasia ranges from almost complete to mild. In severe hypoplasia cases, the iris may be reduced to a little tissue residue only visible with gonioscopy or ultrasound biomicroscopy. In less severe phenotypes, the iris can be present but abnormal in transillumination. Other iris changes include coloboma-like lesions, eccentric pupil, or iris ectropion [1, 3, 26].

*Treatment.* One of the most common treatments of cataract associated with aniridia is phacoemulsification, always bearing in mind the fragility of the lens capsule and the possibility of tear during the surgical procedure. In addition to the possibility of implantation of an intraocular lens, the association of an artificial iris device can also be envisioned [4].

Furthermore, various procedures are being developed to relieve the symptoms associated with the iris hypoplasia. These include coloured contact lenses, corneal tattooing, and implantation of iris artificial devices, such as the black diaphragm intraocular lens and the endocapsular ring [27, 28].

Contact lenses are also frequently suggested for the treatment but patients feel difficulty tolerating them for a long-term treatment, ending up undergoing another procedure. Corneal tattooing is a more ancient and simple method, with little complications associated. However, keratopathy is present in most patients and it limits the procedure [27].

**Figure 5:**
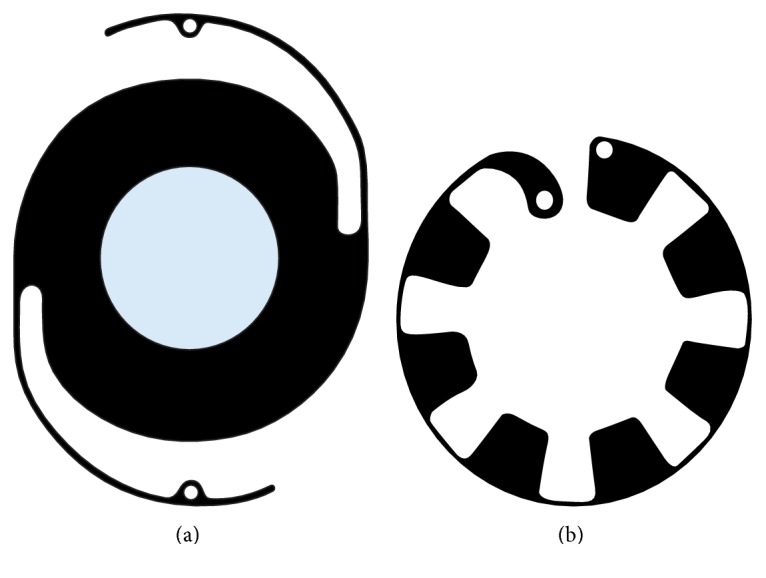
Aniridia Implant Morcher 67F and Aniridia Ring type 50C, respectively (adapted from MORCHER Implants website).

Artificial iris devices with intraocular lens associated or the endocapsular ring are good choices for the treatment of aniridia with associated cataract, as in congenital aniridia. The most used intraocular lenses are Morcher 67F and 67G. The endocapsular ring is type 50C aniridia ring (Figures 5(a) and 5(b)). Morcher lenses are placed on the ciliary sulcus or sutured to the sclera whenever the first choice is not possible due to lack of stability. The aniridia ring was developed to be placed endocapsularly and the simultaneous implantation of two of these rings, in association with an intraocular lens, is needed for them to function as an iris. When choosing the most appropriate device, it is important to know the device's dimensions and consequently the corneal incision length for the placement of the device. Morcher devices need long corneal incisions and with the aniridia ring the incisions are smaller. However, the aniridia ring is fragile and needs the capsule to be present to allow its placement [1, 27, 28].

Artificial iris devices improve the VA, reduce photophobia, decrease the magnitude of the associated nystagmus, and aesthetically improve the eye's appearance. Despite their global safety profile, their implant has some possible complications, such as device fracture, suboptimal placement, capsular tear, or hyphema. After the surgery, the eye may develop prolonged inflammation, uveitis, glaucoma, keratopathy worsening, retinal detachment, migration of the prosthesis, or even endophthalmitis [27, 28].

Artificial personalized iris implants are being developed with the aim of substituting the iris diaphragm. They are made of biocompatible silicone and are coloured accordingly to the contralateral eye's colour. The foldable properties of these devices permit its insertion through a minimal corneal incision sometimes associated with an intraocular lens of any type if necessary [28].

#### 3.3.4. Retina and Optic Nerve

Optic nerve hypoplasia is common in aniridia and it has been documented in approximately 10% of the patients. However, this hypoplasia is difficult to diagnose due to the difficulties experienced in examining the eye fundus because of nystagmus, keratopathy, and lens opacifications. Thus, this condition may be underdiagnosed. Macular and foveal hypoplasias are also associated with aniridia. These three hypoplasias have implication on the VA. In the majority of patients with aniridia, a horizontal pendular nystagmus may occur in association with macular hypoplasia [1, 2, 29].

**Figure 6:**
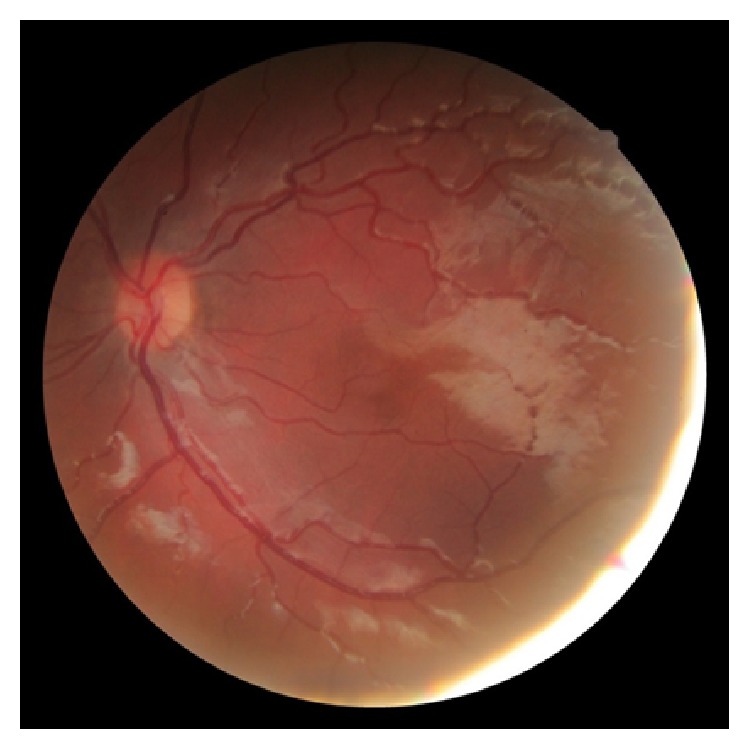
Moderate foveal hypoplasia in patient with aniridia.

Foveal hypoplasia is found in aniridia as well as in albinism but it has also been described as an isolated finding (Figure 6). The normal foveal development starts at the 25th fetal week and it is completely developed between the 15th and the 45th month after birth. Different degrees of foveal hypoplasia can be found depending on the time when the foveal formation is arrested. Different degrees of foveal affectation have different VA affection. Congenital nystagmus may be associated with hypoplasia in aniridia and researchers try to correlate clinical findings with the visual manifestations found in patients. The study of hypoplasia is based on eye fundus examination (foveolar reflex, macular pigmentation, and optic disc observation), optical coherence tomography, and fluorescein angiography (to observe capillary-free zone and the presence of macular pigments). The most frequent findings are reduced foveolar reflex, macular hypopigmentation and retinal vessels infiltrating the fovea [3, 30–32].

Thomas et al. [30] proposed a classification for foveal hypoplasia based on the different stages when foveal development arrests. To classify foveal hypoplasia into grades, they used OCT spectral-domain optical coherence tomography findings which correspond to the various stages of developmental arrest. Firstly, they chose incursion of the plexiform layers into the fovea as the diagnostic criteria of foveal hypoplasia. Then, they proposed the following 4-grade classification:

- grade 1: absence of extrusion of plexiform layers;
- grade 2: grade 1 + absence of foveal pit;
- grade 3: grade 2 + absence of outer segment lengthening;
- grade 4: grade 3 + absence of outer nuclear layer widening.

This classification is an important prognostic value for VA. The hypoplasia grade seems to be inversely related with the best corrected VA.

The etiology and severity of the retinal dysfunction are questionable. Descriptions of the retinal impairment vary and they can go from almost normal to extremely dysfunctional, with various retinal layers involved (as the outer photoreceptor layer or the inner retinal layers). It is also known that rods and cones are equally affected in aniridia. Phototoxicity secondarily to iris hypoplasia is also a factor for greater retinal dysfunction. The use of electroretinography for retinal functional evaluation can be useful in assessing these cases [1].

Aniridia also increases the probability of retinal tears and detachments. There is the high probability of retinal detachment after a tear, even when previous history of cataract extraction or posterior segment surgery is not present [1].

### 3.4. Diagnosis and Genetic Study in Aniridia

Aniridia is firstly suspected at clinical level in a paediatrician visit due to child's visual deficit. An ophthalmologist confirms the diagnosis afterwards. The investigation starts with a search for anomalies of the cornea, iris, and lens and the finding of nystagmus, strabismus, or anterior chamber malformations. It is important to verify the presence of drainage angle anomalies and signs of glaucoma. Eye fundus examination is essential to diagnose foveal, macular, and/or optic nerve hypoplasia. The most important exam is ophthalmoscopy to characterize the iris or pupil anomalies, opacifications and corneal neovascularization, and presence of cataract or glaucoma. Fundoscopic examination characterizes the foveal hypoplasia and optic nerve abnormalities. Optical coherence tomography findings can be used to confirm foveal hypoplasia and to grade it, yet there is the additional difficulty related to the nystagmus [3, 30].

Differential diagnosis includes malformations affecting the anterior segment of the eye (Rieger type I syndrome and Peters syndrome), iris coloboma, albinism (oculocutaneous or ocular), and the Gillespie syndrome. Other causes of infantile nystagmus and VA reduction without iris abnormalities, like retinal dysplasia, congenital cataract, isolated optic nerve hypoplasia, or congenital infections, should also be considered [3].

After the clinical diagnosis, it is important to inquire about the family history. If there is an affected parent, it is unlikely that the child will have deletion extending to the WT1. It is also important to perform an ophthalmologic examination of the parents to look for PAX6 dysfunction related abnormalities even when familial history is negative [3].

Then, a genetic diagnosis of aniridia is conducted to confirm the clinical diagnosis, to evaluate the risk of Wilms tumour, to provide genetic counselling, and to try to determine the evolution and prognosis of mutations with reported genotype-phenotype correlation, although, as was previously explained, this correlation is weak. Most commonly, the clinical confirmation of aniridia is not discarded even when the mutation is not identified. There is a broad range of techniques available to detect mutations in the PAX6 [6].

To detect different types of genetic abnormalities of PAX6 gene, the techniques currently used are [6] as follows:

- unique mutations: screening techniques or DNA sequencing (sensitivity between 19% and 99%);
- deletions: multiplex ligation-dependent probe amplification (MLPA), cytogenetic techniques (fluorescence* in situ* hybridization (FISH), and high resolution karyotype), comparative genomic hybridization array (CGH-array) or family study with general markers.

It is also important to analyse the PAX6 coding region since mutations also exist beyond the encoding sequence of PAX6. This analysis is able to detect 45% to 55% of familiar aniridia cases [6].

## 4. Conclusion

Aniridia is a complex disease affecting the various segments of the eye. The visual prognostic of patients with aniridia is poor from early ages. Most of the patients with aniridia present with foveal and/or macular hypoplasia since birth. The stimulation to the optic nerve is also poor due to the associated nystagmus and the strabismus that are present in many patients. Further on, keratopathy changes develop, as well as the rise of IOP with the consequent development of glaucoma. Cataract may also develop and all these affect the final visual acuity.

Thus, aniridia is a challenging disease and it is important to keep investigating for new approaches to the treatment of these associated disorders that in time appear in aniridia, specially the keratopathy and glaucoma.

## Figures and Tables

**Table 1:** Results of studies with keratoprosthesis.

Authors	No. Patients /number of eyes	Age (years)	Complications (intraoperative /follow-up)	Best Corrected Visual Acuity			Best final corrected visual acuity	Associated procedures /number of patients	% prosthesis retention	Follow-up (months)
				Improvement	Without improvement /worsening	Worsening				

Akpek et al. [10]	15/16	25–66 Median: 50	0/1 glaucoma [GW] + 3 CD + 1 RD [after CD] + 2 RPM + 1 CTM^a^	14	1	0	HM to 20/60 Median: 20/200	LE, IOLE, GDD, PPV/10	100	2–85 Median: 17

Bakhtiari et al. [14]	9/9	11–71 Mean: 33,8	0/1 RD + 1 SH + 5 RPM	9	0	0	2/500 to 20/200	IOLE/7; PPV/9	100	6–48 Mean: 26,1

Rixen et al. [15]	7/7	12–85 Mean: 52	0/1 glaucoma [GW] + 1 WD + 3 RPM	6	0	1^b^	LP to 20/100 Median: 20/200 (with spectacles)	GDD/4; GDD revision/1; LE/1; IOLE/2; PPV/3	100	3–30 Median: 18

Greiner et al. [16]^c^	4/5 (35/40)	(2–86 Mean: 52,9)	(0/22 RPM + 34 glaucoma + 5 endophthalmitis + 6 CTM + 6 keratoprosthesis extrusion)	2	3		2 eyes maintained VA ≥ 20/200; 2 eyes had initial VA improvement that was not substantial (did not maintain VA ≥ 20/200); 1 eye never achieved VA ≥ 20/200	(LE/13; pupilloplasty/1; tarsorrhaphy/1; GDD/3; AMT/1; AV/1)	80 (80)	(5–72 Mean: 33,6)

Kang et al. [17]^cd^	5/5 (19/21)	(27–83 Mean: 51,9)	(0/8 glaucoma [3 GW + 5 IOPE + 3 CD + 2 PED + 1 endophtalmitis + 7 MO + 3 VH + 2 EM + 10 RPM + 1 CTM + 2 LLR)	(19)	(2)		(6 patients—≤20/200; 11 patients—≥20/200; 4 patients—≥20/50)	(LE/14; iridectomy/7; IOLE/3; tarsorrhaphy/3; GDD/2; AV/8; PPV/3)	80^e^ (90,5)	(6–36, 3 Mean: 14,6)

AMT (amniotic membrane transplant); AV (anterior vitrectomy); CD (choroidal detachment); CTM (corneal tissue melt); EM (epiretinal membrane); GDD (glaucoma drainage device implantation); GW (glaucoma worsening); HM (hand movement); IOLE (intraocular lens extraction); IOPE (intraocular pressure elevation); LE (lens extraction or cataract removal); LLR (lower lid retraction); LP (light perception); MO (macular oedema); PED (persistent epithelial defects); PPV (pars plana vitrectomy); RD (retinal detachment); RPM (retroprosthetic membrane); SH (suprachoroidal hemorrhage); VH (vitreous hemorrhage); WD (wound dehiscence).

^a^This patient received an older type of the keratoprosthesis which justified that complication. After that he had a scleral patch reinforcement of the prosthesis and there was no need to extract the device neither affectation of VA.

^b^Initially, the patient had VA improved with BCVA 20/300 with spectacles but suffered a massive occipital hemorrhagic stroke which the authors point to be the reason for the final VA (light perception only).

^c^This study used patients with keratopathy who had different diagnoses (including patients with aniridia). When specific data for aniridic patients was available, it is shown in the table. Otherwise, data from the overall patients is used and is specified between brackets.

^d^In this study, the keratoprosthesis was used as the primary penetrating corneal procedure.

^e^The aniridic patient who had not retained the prosthesis developed corneal tissue melt surrounding the device as a complication. Further on he had two more replacement procedures.
